# Revealing the Boundaries of Selected Gastro-Intestinal (GI) Organs by Implementing CNNs in Endoscopic Capsule Images

**DOI:** 10.3390/diagnostics13050865

**Published:** 2023-02-23

**Authors:** Sofia A. Athanasiou, Eleftheria S. Sergaki, Andreas A. Polydorou, Alexios A. Polydorou, George S. Stavrakakis, Nikolaos M. Afentakis, Ioannis O. Vardiambasis, Michail E. Zervakis

**Affiliations:** 1 School of Electrical and Computer Engineering, Technical University of Crete, 73100 Chania, Greece; 2 Department of Electronic Engineering, Hellenic Mediterranean University, 73133 Chania, Greece; 3 Aretaeio Hospital, 2nd University Department of Surgery, Medical School, National and Kapodistrian University of Athens, 11528 Athens, Greece; 4 Medical School, National and Kapodistrian University of Athens, 11528 Athens, Greece

**Keywords:** computer-aided detection, wireless endoscopic capsule, gastro-intestinal, CNN, artificial intelligence (AI) GI organ boundary, capsule tracking

## Abstract

Purpose: The detection of where an organ starts and where it ends is achievable and, since this information can be delivered in real time, it could be quite important for several reasons. For one, by having the practical knowledge of the Wireless Endoscopic Capsule (WEC) transition through an organ’s domain, we are able to align and control the endoscopic operation with any other possible protocol, i.e., delivering some form of treatment on the spot. Another is having greater anatomical topography information per session, therefore treating the individual in detail (not “in general”). Even the fact that by gathering more accurate information for a patient by merely implementing clever software procedures is a task worth exploiting, since the problems we have to overcome in real-time processing of the capsule findings (i.e., wireless transfer of images to another unit that will apply the necessary real time computations) are still challenging. This study proposes a computer-aided detection (CAD) tool, a CNN algorithm deployed to run on field programmable gate array (FPGA), able to automatically track the capsule transitions through the entrance (gate) of esophagus, stomach, small intestine and colon, in real time. The input data are the wireless transmitted image shots of the capsule’s camera (while the endoscopy capsule is operating). Methods: We developed and evaluated three distinct multiclass classification CNNs, trained on the same dataset of total 5520 images extracted by 99 capsule videos (total 1380 frames from each organ of interest). The proposed CNNs differ in size and number of convolution filters. The confusion matrix is obtained by training each classifier and evaluating the trained model on an independent test dataset comprising 496 images extracted by 39 capsule videos, 124 from each GI organ. The test dataset was further evaluated by one endoscopist, and his findings were compared with CNN-based results. The statistically significant of predictions between the four classes of each model and the comparison between the three distinct models is evaluated by calculating the *p*-values and chi-square test for multi class. The comparison between the three models is carried out by calculating the macro average F1 score and Mattheus correlation coefficient (MCC). The quality of the best CNN model is estimated by calculations of sensitivity and specificity. Results: Our experimental results of independent validation demonstrate that the best of our developed models addressed this topological problem by exhibiting an overall sensitivity (96.55%) and specificity of (94.73%) in the esophagus, (81.08% sensitivity and 96.55% specificity) in the stomach, (89.65% sensitivity and 97.89% specificity) in the small intestine and (100% sensitivity and 98.94% specificity) in the colon. The average macro accuracy is 95.56%, the average macro sensitivity is 91.82%.

## 1. Introduction

All the recent hardware and software advances incorporated in the technology of Wireless Endoscopic Capsule (WEC) robots constantly demonstrate their great potential for improvement, as research boundaries progress even further, revealing new opportunities for gastroenterologists and patients, for better diagnosis and treatment. The WEC has the potential to become a screening, diagnostic and therapeutic technique for the entire GI tract because of its low discomfort to users [1,2,3,4]. Thus, the WEC has become the first choice for small bowel endoscopy in children [5,6,7]. Moreover, the use of a robotic WEC (in the future) promises to offer some form of targeted “smart” therapy, e.g., drug delivery, clip release for bleeding control, precise biopsy sampling, etc. Nevertheless, there are some specific computer-aided techniques based on AI that are approved by the European Medicines Agency (EMA) and by the Pharmaceuticals and Medical Devices Agency (PMDA) since 2018 [8]:

- CADe by FUJIfilm, PENTAX,
- GI Genious by Medtronic,
- EndoBRAIN by Cybernet,
- Endo-AID by Olympus (as of this time, pending approval).

To the best of our knowledge, no methods or algorithms seem to have been proposed yet to automatically detect the capsule passing by selected GI organs (their extend, from start to finish), although medical reviewers can surely identify such a position through their optical review and diagnosis later on. This kind of automation can play a significant role in the near future, as technology evolves rapidly towards better, cheaper and more efficient endoscopic capsules. The present work reveals such a case, specifically the detection of WEC’s transition and regional awareness while passing the boundaries of gastrointestinal (GI) organs through the GI tract.

A lot of algorithms are proposed for reducing WEC’s videos review time by the experts, by detecting and diagnosing of hemorrhage and GI tract lesions based on AI [9,10,11,12]. The topic of localizing the capsule inside GI has gained a lot of interest among researchers. Few robotic research groups have proposed methods for the real time localization of the capsule, possibly by providing wireless endoscopic capsule control through magnetic devices and other RF localization techniques [13,14].

To the best of our knowledge, the most relevant algorithms capable of classifying single frame images into GI organs, or detecting the boundaries of the GI organs are presented in references [15,16]. In [15], a CNN algorithm with a temporal filtering detects three GI organs (the stomach, the small bowel, and the colon), but is not able to identify the boundary transitions among these organs. On the other hand, the algorithm of [16], employing the long-term dependencies of WEC frames, detects both transitions between stomach and small bowel and between small bowel to colon. Moreover, the CAD tool based on CNN and SVM algorithms for narrow band imaging endoscopy is implemented into the FPGA-based prototyping platform [17].

In this study, the problem to solve is the real-time tracking of the WEC transition through the boundaries of GI’s organs (esophagus to stomach, stomach to small intestine, small intestine to colon), from CNN multi-class classification of endoscopy capsule images. Moreover, the algorithms can be executed from a mobile platform that can be physically attached for the time of recording upon the patient. Another aim is to study how the difference of the kernel size and number effects the CNN’s performance or how adding dropout layers will affect the possibility of overfitting and underfitting of the proposed CNN classifier.

## 2. Materials and Methods

This prospective, noninterventional study was conducted at Aretaeio Hospital, at the Department of Surgery, School of Medicine, National and Kapodistrian University of Athens, Athens, Greece from December 2019 to June 2020. The School of Electrical and Computer Engineering of the Technical University of Crete, Greece and the Department of Electronic Engineering, Hellenic Mediterranean University, were responsible for CAC algorithm development.

### 2.1. Data and Resources

For this research, we have used video data out of a total of 138 PillcamTM SB endoscopic capsule videos collected from random anonymous patients hospitalized in four different Gastroenterology Departments of (a) the Medical School of the National and Kapodistrian University of Athens, (b) the Attikon University Hospital, (c) the Laikon University Hospital and (d) the Medical School of the Aristotle University of Thessaloniki. All these 138 patients have been diagnosed by their attending doctors positive to: (i) bleeding, (ii) angiodysplasias, (iii) haemangiomas, or (iv) lesions which predispose to bleeding in the small intestine. The 138 videos have been evaluated by the third coauthor, who is a very experienced endoscopist, and divided into two sets: the training set with 99 videos which are used by the proposed algorithm for feature extraction and the validation set with the rest of the 39 videos which are used for independent validation as images unseen from the algorithm. From those videos, there was an extraction of total 5520 frames, 1380 frames for each organ (ten frames for each organ boundary of each video) of interest: esophagus, stomach, small intestine, colon. In order to achieve success in regional information extraction of the endoscopy capsule, the problem to be solved is the organ’s entrance (gate) classification.

**Figure 1:**
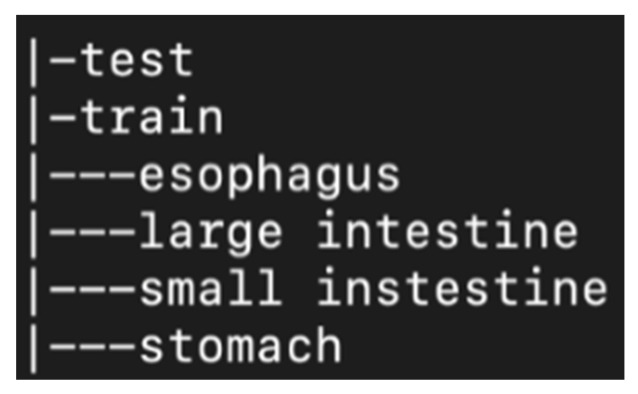
Folder structure.

**Figure 2:**
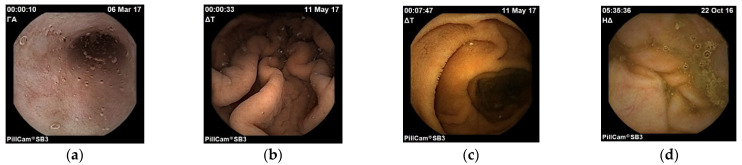
Images of esophagus (**a**), stomach (**b**), small intestine (**c**) and colon (**d**).

After extracting the images in order to use them for machine learning purposes, images were divided in different folders. First of all, they were distinguished in training data (total 5520 frames, 1380 for each organ) and in testing data (total 496 frames, 124 frames for each organ). Training data are the ones that are fed into the model in the training phase and testing are the ones used in order to verify that the model is producing comprehensive training results. Afterwards, the training data were split in four folders (see Figure 1). Each folder represents one of the four points of interest. For example, the stomach folder contains only images that are coming from that area. Moreover, for the training process, a CSV file was created in which there is a labelling of the images based on the category they belong to and their name. In Figure 2, images samples are shown.

All images are in three colour channels (RBG—Red Blue Green), with the typical pixel level [0, 255], although the data are normalized during the experiment. The data of the training folder are separated so that 80% of them are used for the phase of training the model. The rest of the 20% are used as validation data during the training process. Validation data are important so that we have a quicker and better feedback on the model and we do not fall into cases of overfitting or underfitting [18].

To conclude, before feeding the data into the training phase of the model, the following actions were performed:

- create validation data using 20% of the training data, in order to be used as unknown data during the training process;
- reduce the size of images from the 576 × 576 original image size to 256 × 256;
- define the batch size to be 16;
- use autotune data, in order to help our model to be faster, since it will decouple the time when data is produced from the time when data is consumed. It will use a background thread and an internal buffer to prefetch elements from the input dataset ahead of the time they are requested. Autotune function will prompt the tf.data to tune the value dynamically at runtime;
- perform data normalization, by transforming all pixel values in the [0, 1] interval.

### 2.2. Proposed CNNs 4-Class Classifiers

Taking under consideration similar research that classifies images into corresponding categories, the Convolutional Neural Network (CNN) model is well known to be good at image classification. CNN stands for Convolutional Neural Network, where each image goes through a series of convolution and max pooling for features extraction. CNN takes an input image and assigns importance (weights and biases) to various features to help in distinguishing images, for example, animal classification [19], bleeding and non-bleeding images in endoscopy capsule [11], ImageNET [20], concluded that Convolutional Neural Networks were a great starting point.

In general, CNN contains a series of connected layers, containing the convolutional layer, the pooling layer and the dense layer. For this research, three different CNN models were created and evaluated. Additionally, there was a testing of those models including data augmentation. The input of our models at all times are images in three color channels (RGB) with final dimensions [256 × 256]. Then, the pixel values were normalized. This is achieved by subtracting the mean of each pixel and then diving that result by the standard deviation. That will lead to a Gaussian curve centred to zero. In case of images, the possible values are only the positive ones so it will move the data from the value range of [0, 255] to the value range of [0, 1].

The idea around Convolutional Neural Networks is that there are the layers that will help in feature extraction, the convolutional layers in cooperation with the pooling layers, and the layers that will transform their input to a decision, which are the flatten layers. Convolutional layers will perform a linear matrix multiplication by sliding the filter windows through the image pixels.

In our research, when it comes to the convolutional layers in Model 1 and 2, the filters are of the size [3 × 3], and in Model 3 are of the size [5 × 5]. From layer to layer in each model, what is changed, also, is the number of filters applied for example, in Model 1 (Figure 2), firstly, we apply 32 filters, later on 64 and finally 128. Between the Convolutional Layers there is a pooling function, that function helps in order to select the most powerful features, in a window of [2 × 2]; it will parse the outcome of the convolutional layer and select the maximum values. In the next connected layer, the filters will be applied to the strongest feature in order to extract the ones that are more relevant. This will provide also the advantage of downsampling the features and reducing the computational cost of the algorithm and the same time we will care for the most relevant characteristics of the images.

For the code and result production, open source tools were used. All code was written in Python and run in the Google Colab platform in order to have an environment with more resources in memory and GPU than the local system. The libraries that were used for data pre-processing and model creation are Tensorflow and Keras. For image manipulation, the cv2 library was used. From Keras, in order to create the models’ layers, we used the functions: Conv2D, Maxpool2D, Flatten and Dense, Sequential (in order to define that we are referring to a Convolutional Neural Network).

#### 2.2.1. CNN–Model 1

**Figure 3:**
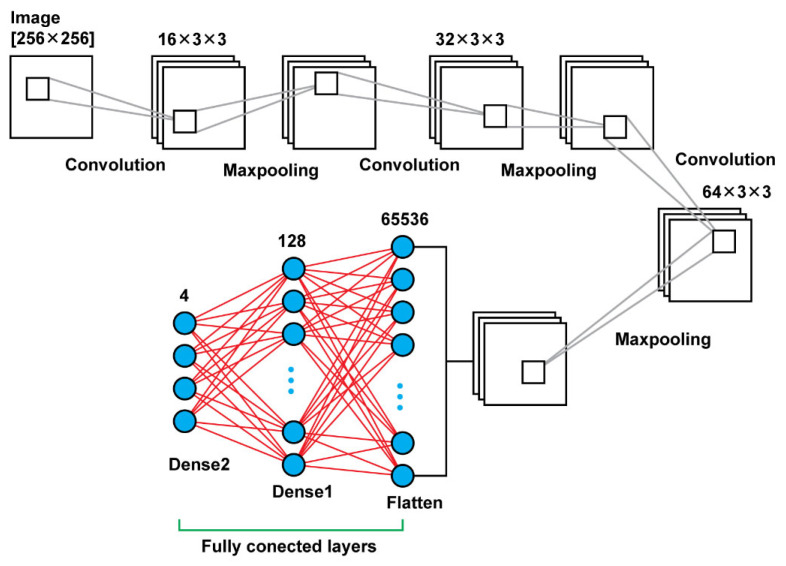
Model 1 architecture.

Model 1 consists connected two dimensional convolution layers and two dimensional maxpooling layers as feature extractors, a flatten layer in order to have a vector of features after the last pooling layer that will be an input for the upcoming two dense layers that follow in order to make the final decision of the classification. In total, using that model, there were 8,412,836 trainable parameters. In Table 1 are shown the details regarding the different layers as well as the sizes of the filters and the trainable parameters. In Figure 3, there is a graphical representation of Model 1.

#### 2.2.2. CNN–Model 2

**Figure 4:**
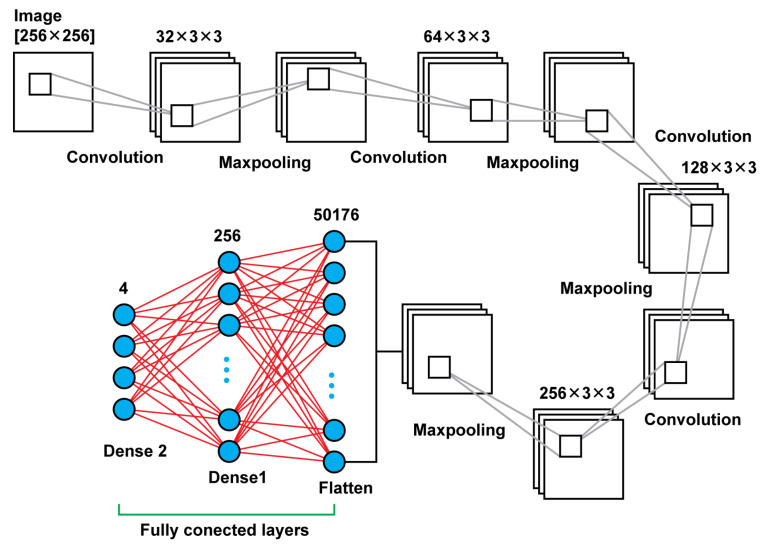
Model 2 architecture.

Model 2 is a deeper neural network using, again, connected two dimensional convolution layers and two dimensional maxpooling layers as feature extractors, a flatten layer in order to create a vector of features after the last pooling layer that will be an input for the upcoming two dense layers. In total, that model leads to 13,235,756 trainable parameters. In Table 2, there are details regarding output size of the different layers as well as the sizes of the filters and the trainable parameters. In Figure 4, there is a graphical representation of Model 2.

#### 2.2.3. CNN–Model 3

**Figure 5:**
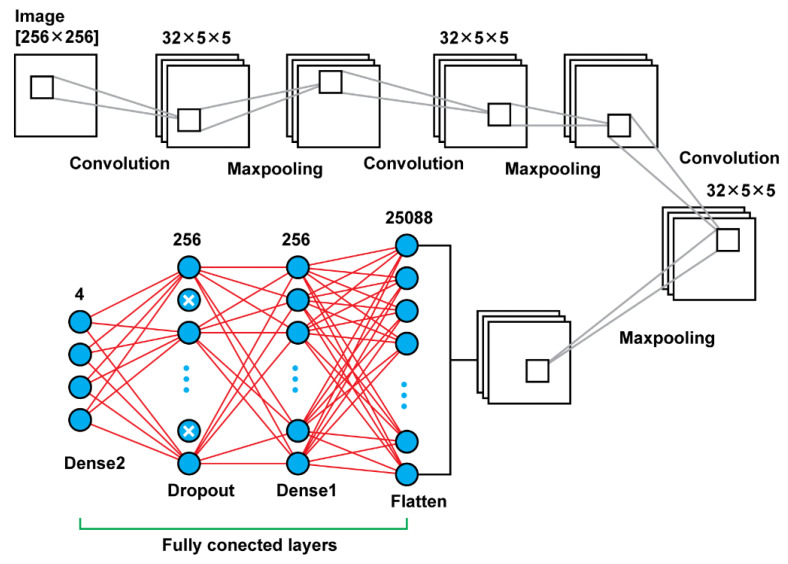
Model 3 architecture.

Model 3 is using convolutional layers with filters of the size (5 × 5) instead the filters of size (3 × 3) that were using the previous models. Moreover, in the previous models, there was an increase of the number of filters that were used, as the network was getting deeper. For this model, there were used in all steps filters of the same size and number; in every convolutional layer there were used 32 filters of the size (5 × 5). Another difference of this model is that there was used also a dropout layer between the last two dense layers. Other than those two key differences, the rest remain the same. In total, that model results in 6,477,508 trainable parameters. In Figure 5, there is a graphical representation of Model 3 and Table 3 presents the details.

## 3. Results of CNNs Training

Taking under consideration the relatively small set of data (5520 frames selected from 99 videos) that we are using for training as well as the fact that the data did not undergo any pre-processing before arriving at the training process, our results stand out. There are references on the curves that refer to the loss and accuracy of training and validation data as per epoch of the training process. Those curves are provided as per model.

All models used 30 epochs in order to conclude to a stable value of validation and training accuracy. This amount of epochs worked well enough to allow our training model to conclude with a good training convergence. There was used also instead of individual images, a patch of images of size 16. For the Model 1, training accuracy and validation accuracy start being stable after epoch 15; for Model 2 we see the same behavior after epoch 20 and for the Model 3, those values start being stable after epoch 25. We observed, after a relatively small amount of epochs, the results we were taking were quite sufficient. Model 1 concludes with training accuracy 1 and validation accuracy 0.9677. Model 2 provides training accuracy 1 and validation accuracy 0.9677. Moreover, Model 3 provides training accuracy 1 and validation accuracy 1. All those values refer to the results of the 30th epoch. Among the three models, the one that provides a more stable view, as many times as the experiment of training was reproduced, is Model 2. We observed that increasing the number of filters as the model becomes deeper shows that is providing a more stable result.

## 4. Methods of Multiclass CNNs Independent Evaluation

The independent evaluation for the prediction quality of our multiclass CNNs, is the trained models test, examining data not used in the training procedure. To evaluate the quality of the performance of any multiclass classifier and compare them to each other, it is broken into multiple binary classification models. The Mattheus correlation coefficient (MCC), calculates a high score only if the prediction obtained correct results in all of the confusion matrix categories (true positives, false negatives, true negatives, and false positives), proportionally both to the size of positive elements and the size of negative elements in the dataset [21].

(1) $$MCC=\frac{c\times s-\sum_{k}^{K}p_{k}\times t_{k}}{\sqrt{\left(s^{2}-\sum_{k}^{K}p_{k}^{2}\right)\times \left(s^{2}-\sum_{k}^{K}t_{k}^{2}\right)}}$$

where *K* is the number of classes,

- $t_{k}=\sum_{i}^{K}C_{ik}$ is the number of times class *k* truly correct,
- $p_{k}=\sum_{i}^{K}C_{ik}$ is the number of times class *k* was predicted into class,
- $c=\sum_{k}^{K}C_{kk}$ is the total number of samples that was correctly predicted,
- $s=\sum_{i}^{K}\sum_{j}^{K}C_{ij}$ is the overall number of samples, and
- $c\times s-\sum_{k}^{K}p_{k}\times t_{k}$ includes the elements wrongly classified by the model and covers multiplicative entities that are weaker than the product *c* × *s*.

To evaluate each multi class classifier, the macro average *F*1 score indicates how the classifier performs for every class [22]. The macro average involves computing one versus all confusion matrices for each class, where each individual *i*th class is the positive class and all the other classes are the negative class. Since our experiment refers to a 4-class classification, for each class we calculate the values of True Positive (*TP*), True Negative (*TN*), False Positive (*FP*), False Negative (*FN*). The macro average *F*1 score is defined by the harmonic mean of the macro average of recall (or sensitivity) and macro average precision as:

(2) $$F1_{macro}=2\frac{P_{macro}\times R_{macro}}{P_{macro}+R_{macro}}$$

where the macro average calculates a simple average of the binary classification metric values of all classes,

(3) $$P_{macro}=\frac{1}{K}\sum_{i=1}^{K}P_{i}=\frac{1}{K}\sum_{i=1}^{K}\frac{TP_{i}}{TP_{i}+FP_{i}},$$

(4) $$R_{macro}=\frac{1}{K}\sum_{i=1}^{K}R_{i}=\frac{1}{K}\sum_{i=1}^{K}\frac{TP_{i}}{TP_{i}+FN_{i}},$$

where precision of the *i*th class, *P_i_*, stands for the fraction of positive class predictions that were actually positive. The value of sensitivity or recall of the *i*th class, *R_i_*, stands for the fraction of all positive samples that were correctly predicted as positive by the classifier.

*Accuracy* is providing the overall accuracy of a model for a specific class, meaning the fraction of the total class objects that were correctly classified by the classifier. The accuracy of predicting the frames that refer to a class of a model is calculated by Equation (5):

(5) $$Accuracy=\frac{TP+TN}{TP+TN+FP+FN}$$

Furthermore, *Error Rate*, which indicates the quality of the classifiers towards the wrong predictions, is calculated by Equation (6):

(6) $$Error\;Rate=\frac{FP+FN}{TP+TN+FP+FN}$$

and *Specificity*, which presents the fraction of all negative samples that are correctly predicted as negative by the classifier, is calculated by Equation (7):

(7) $$Specificity=\frac{TN}{TN+FP}$$

In the present testing, we evaluate the statistical significance of predictions between the three CNN models by calculating the test result for *p*. If the *p*-value is greater than 0.05 then no effect is observed, meaning that our test hypothesis is false and the outcomes are statistically independent. Our null hypothesis is that all the models are equivalent, so “Ho = there is no difference between the models”.

The basic format for reporting a chi-square test result is:

x^2^ (degrees of freedom, *N* = sample size) = chi-square statistic value, *p* = *p* value, while our *p*-values are calculated by the Pearson’s chi-squared test, using Equation (8):

(8) $$x^{2}=\sum_{i=1}^{r}\sum_{j=1}^{c}\frac{{\left(O_{i,j}-E_{i,j}\right)}^{2}}{E_{i,j}}$$

where *r* is the number of rows corresponding to the number of models, *c* is the number of columns corresponding to the number of classes of the contingency table, *df* is the degrees of freedom calculated by *df* = (*r* − 1) × (*c* − 1) for test of independence, *E_i,j_* is the “theoretical frequency” for a cell, given the hypothesis of independence, and *O_i,j_* is the observations of type *j* ignoring the row attribute (fraction of column totals).

## 5. Results of CNN Models Testing

After using the formulas above for each class versus all classes and for each model, the results of the metrics when it comes to each model are presented in Table 4, Table 5, Table 6 and Table 7. For Model 1, the report is in Table 4 and its performance analysis in Table 7. We can observe that the model is working well for all organs, with slightly bigger error rate when it comes to the stomach. After the examination of the error that we saw in the predictions, many times this error occurs because the presence of pituitary or of saliva is misunderstood as the pituitary of the colon which is causing confusion in the model.

Next, for Model 2, the report in Table 5 and its performance analysis in Table 7 show that this model is the most accurate among the three, when it comes to predictions; since the error rate is improved for stomach frames prediction and it also provides the best metrics for esophagus frames prediction.

Lastly, the Model 3 report and performance metrics appear in Table 6 and Table 7. As we can observe, Model 3 provides the biggest error rate in stomach frames prediction among the rest of the models. Although, the predictions for esophagus and small intestine frames have better metrics using that model.

Our results of independent validation and cross validation, demonstrate that the best of our three distinct models is that of Model 2.

For the 4 × 4 contingency table of each of our CNN1, CNN2 and CNN3 models, our chi-square test results separately in a *p*-value ≦ 0.00001, showing that the four-class classification of each model is statistically independent.

Moreover, for the 3 × 4 contingency table comparing our three CNN models, the chi-square test results in a *p*-value ≦ 0.00001. It is showing that the outputs of the three four-class models are statistically independent.

## 6. Conclusions

By using our research as a tracking and four classes classification algorithm, physicians can successfully detect the gates of the four GI organs (and, furthermore, to recognize the digestive organ) that the capsule is passing by, in real time. The algorithms can be executed from a mobile platform that can be physically attached for the time of recording upon the patient. This can be used as a passive compass while navigating through the digestive tract, to reveal better metrics concerning each capsule design potential (i.e., to overcome palindrome movements or to obtain better insight on how to cope through them), as well as an activation control tool for a number of actions. The whole procedure will provide better accuracy per individual patient (in terms of charting topological idiosyncrasies), without any added cost (same hardware, the processing power needed should be provided by an external unit linked to the capsule). With the future advancement of technology, when sufficient electrical power will be available to ensure the independence of the capsule unit, it will become possible to embed in the capsule a microprocessor to execute the algorithm. For now, the algorithm could be executed upon an external mobile unit, as the video frames are transferred wirelessly in real time.

## Figures and Tables

**Table 1:** Model 1 summary.

Layer Name	Layer Type	Number of Filters	Output Shape	Number of Parameters
Conv2D	Conv2D	16	(256, 256, 16)	448
Max_pooling2D	MaxPooling2D		(128, 128, 16)	0
Conv2D_1	Conv2D	32	(128, 128, 32)	4640
Max_pooling2D_1	MaxPooling2D		(64, 64, 32)	0
Conv2D_2	Conv2D	64	(64, 64, 32)	18,496
Max_pooling2D_2	MaxPooling2D		(32, 32, 64)	0
Flatten	Flatten		65,536	0
Dense	Dense		128	8,388,736
Dense_1	Dense		4	516

**Table 2:** Model 2 summary.

Layer Name	Layer Type	Number of Filters	Output Shape	Number of Parameters
Conv2D	Conv2D	32	(254, 254, 32)	896
Max_pooling2D	MaxPooling2D		(127, 127, 32)	0
Conv2D_1	Conv2D	64	(125, 125, 64)	18,496
Max_pooling2D_1	MaxPooling2D		(62, 62, 64)	0
Conv2D_2	Conv2D	128	(60, 60, 128)	73,856
Max_pooling2D_2	MaxPooling2D		(30, 30, 128)	0
Conv2D_3	Conv2D	256	(28, 28, 256)	295,168
Max_pooling2D_3	MaxPooling2D		(14, 14, 256)	0
Flatten	Flatten		50,176	0
Dense	Dense		256	12,845,312
Dense_1	Dense		4	1028

**Table 3:** Model 3 summary.

Layer Name	Layer Type	Number of Filters	Output Shape	Number of Parameters
Conv2D	Conv2D	32	(252, 252, 32)	2432
Max_Pooling2D	MaxPooling2D		(126, 126, 32)	0
Conv2D_1	Conv2D	32	(122, 122, 32)	25,632
Max_Pooling2D_1	MaxPooling2D		(61, 61, 32)	0
Conv2D_2	Conv2D	32	(57, 57, 32)	25,632
Max_Pooling2D_2	MaxPooling		(28, 28, 32)	0
Flatten	Flatten		25,088	0
Dense	Dense		256	6,422,784
Dropout	Dropout		256	0
Dense_1	Dense_1		4	1028

**Table 4:** Report from testing 496 frames (124 not seen before frames of each organ) with CNN model 1.

Matrix C			Predicted Values				
	Class *k*		*TP_ii_*	*TN*	$\sum_{i=1}^{4}FP_{i}\;$	$\sum_{i=1}^{4}FN_{i}\;$	Total *t_k_*
Actual values	**1**	**Esophagus**	*C*_11_ = 27	87	8	2	*t_k=_*_1_ = 124
	**2**	**Stomach**	*C*_22_ = 25	83	4	12	*t_k=_*_2_ = 124
	**3**	**Small Intestine**	*C*_33_ = 26	91	4	3	*t_k=_*_3_ = 124
	**4**	**Colon**	*C*_44_ = 29	94	1	0	*t_k=_*_4_ = 124
	**Total *p_k_***		***c =* 107**	***p_k=_*_2_*=* 355**	***p_k=_*_3_*=* 17**	***p_k=_*_4_*=* 17**	***s* = 992**
4 × 4 contingency table Significance Level: 0.05, X^2^ (*N* = 124) = 249.92, *p* < 0.00001. Significant at *p* < 0.05.							

**Table 5:** Report from testing 496 frames (124 not seen before frames of each organ) with CNN model 2.

**Matrix C**			**Predicted Values**				
	**Class *k***		***TP_ii_***	***TN***	$\sum_{i=1}^{4}FP_{i}$	$\sum_{i=1}^{4}FN_{i}$	**Total *t_k_***
Actual values	**1**	**Esophagus**	*C*_11_ = 28	90	5	1	*t_k=_*_1_ = 124
	**2**	**Stomach**	*C*_22_ = 30	84	3	7	*t_k=_*_2_ = 124
	**3**	**Small Intestine**	*C*_33_ = 26	93	2	3	*t_k=_*_3_ = 124
	**4**	**Colon**	*C*_44_ = 29	94	1	0	*t_k=_*_4_ = 124
	**Total *p_k_***		***c =* 113**	***p_k=2_ =* 361**	***p_k=3_ =* 11**	***p_k=4_ =* 11**	***S* = 992**
4 × 4 contingency table Significance Level: 0.05, X^2^ (*N* = 124) = 219.76, *p* = 0.00001. Significant at *p* < 0.05.							

**Table 6:** Report from testing 496 frames (124 not seen before frames of each organ) with CNN Model 3.

Matrix C			Predicted Values				
	Class *k*		*TP_ii_*	*TN*	$\sum_{i=1}^{4}FP_{i}$	$\sum_{i=1}^{4}FN_{i}$	Total *t_k_*
Actual values	**1**	**Esophagus**	*C*_11_ = 27	95	0	2	*t_k=_*_1_ = 124
	**2**	**Stomach**	*C*_22_ = 25	84	3	12	*t_k=_*_2_ = 124
	**3**	**Small Intestine**	*C*_33_ = 22	95	0	7	*t_k=_*_3_ = 124
	**4**	**Colon**	*C*_44_ = 29	77	18	0	*t_k=_*_4_ = 124
	**Total *p_k_***		***c =* 103**	***p_k=_*_2_*=* 351**	***p_k=_*_3_*=* 21**	***p_k=_*_4_*=* 21**	***s* = 992**
4 × 4 contingency table Significance Level: 0.05, X^2^ (*N* = 124) = 223.19, *p* = < 0.00001. Significant at *p* < 0.05.							

**Table 7:** Performance metrics of our CNN models 1, 2 and 3 for independent validation (data not seen before).

Model/Class	Accuracy	Error Rate	Precision	Specificity	Sensitivity (Recall)	*F*1 Score	MCC
Model 1/Class 1 vs. rest	0.9193	0.0806	0.7714	0.9157	0.9310		
Model 1/Class 2 vs. rest	0.8709	0.1290	0.8620	0.9540	0.6756		
Model 1/Class 3 vs. rest	0.9435	0.0564	0.8666	0.9578	0.8965		
Model 1/Class 4 vs. rest	0.9919	0.0080	0.9666	0.9894	1		
Model 1/**Average macro**	**0.9314**	**0.0685**	**0.8667**	**0.9542**	**0.8758**	**0.871188**	**62.53618**
Model 2/Class 1 vs. rest	0.9516	0.0483	0.8484	0.9473	0.9655		
Model 2/Class 2 vs. rest	0.9193	0.0806	0.9090	0.9655	0.8108		
Model 2/Class 3 vs. rest	0.9596	0.0403	0.9285	0.9789	0.8965		
Model 2/Class 4 vs. rest	0.9919	0.0080	0.9666	0.9894	1		
Model 2/**Average macro**	**0.9556**	**0.0443**	**0.9131**	**0.9703**	**0.9182**	**0.915655**	**69.9200**
Model 3/Class 1 vs. rest	0.9838	0.0161	1	1	0.9310		
Model 3/Class 2 vs. rest	0.8790	0.1209	0.8928	0.9655	0.6756		
Model 3/Class 3 vs. rest	0.9435	0.0564	1	1	0.7586		
Model 3/Class 4 vs. rest	0.8548	0.1451	0.6170	0.8105	1		
Model 3/**Average macro**	**0.9153**	**0.0846**	**0.8775**	**0.9440**	**0.8413**	**0.858994**	**57.63078**

## Data Availability

The data used in this study are available on request from the corresponding authors.
